# Assessment of potential factors associating with costs of hospitalizing cardiovascular diseases in 141 hospitals in Guangxi, China

**DOI:** 10.1371/journal.pone.0173451

**Published:** 2017-03-16

**Authors:** Li-fang Zhou, Mao-xin Zhang, Ling-qian Kong, Jun-jun Liu, Qi-ming Feng, Wei Lu, Bo Wei, Lue Ping Zhao

**Affiliations:** 1 Guangxi Medical University, Nanning, Guangxi, China; 2 Fourth Affiliated Hospital of Guangxi Medical University, Liuzhou, Guangxi, China; 3 Fred Hutchinson Cancer Research Center and University of Washington, Seattle, Washington, United States of America; 4 Health and Family Planning Commission of Guangxi, Nanning, Guangxi, China; Shanghai Diabetes Institute, CHINA

## Abstract

**Background:**

The rising cost of healthcare is of great concern in China, as evidenced by the media features negative reports almost daily. However there are only a few studies from well-developed cities, like Beijing or Shanghai, and little is known about healthcare costs in rest of the country. In this study, we use hospitalization summary reports (HSRs) from admitted cardiovascular diseases patients in Guangxi hospitals during 2013–2016, and we investigate temporal trends of healthcare costs and associated factors.

**Methods:**

By generalized additive model, we compute temporal trends of cost per stay (CPS), cost per day (CPD) and others. We then use generalized linear models to assess which factors associate with CPS and CPD.

**Findings:**

Using a total of 760,000 HSRs, we find that CPS appears to be stabilized around $1040 until the middle of year 2015, before exhibiting a downward trend. Similarly, CPD exhibits similar stable pattern. Meanwhile, surgery-specific CPS showed an increase in year 2013–2014, and then stabilized. Drug costs account for over 1/3 of CPS, but they are gradually declining. Costs associated with physicians’ and nurses’ services represent less than 5% of CPS.

We found that age, sex, marital status, occupation and payment methods are significantly associated with CPS or CPD. Interestingly, we found no association between patient ethnicity and these costs. However, we did find that minority patients use more secondary hospitals than Han patients.

**Interpretations:**

Healthcare costs in Guangxi are stable, contrary to the rise portrayed by Chinese mass media. Several factors can be associated with healthcare costs, and these may be useful for developing evidence-based policies. In particular, there is a need to encourage more Han patients to seek care in primary and secondary hospitals.

## Introduction

Biotechnology innovations and economic development worldwide is accompanied by the steadily increasing cost of healthcare, a challenge facing both developed and developing countries[1]. Without effective management strategies, rising healthcare costs could directly impact political and social stability, especially in developing countries like China. Addressing costs is a focal point of recent healthcare reform efforts launched by the Chinese government in 2009. The reform implemented a series of healthcare policies in recent years to curtail rising costs. Among implemented policies is the adoption of generic medication list in August, 2009, including 307 generic drugs and then subsequently broadened to 520 medicines in 2012. The primary goal is to reduce drug costs through the centralized drug purchase program. From the official source, many reform policies have yielded positive results. (http://www.nhfpc.gov.cn/). Aside official reports or experts’ opinions, there lack actual evidences to demonstrate if such policies have effectively controlled healthcare costs. One recent study examined hospitalization costs from 2009–2013 for three large general hospitals affiliated with Peking Universities, and showed that costs of hospitalizations are gradually increasing, without adjusting for consumer price index (CPI) [2]. However, since CPI of China has been rapidly increasing in recent years, the study adjusted hospitalization costs to the CPI and found that CPI-adjusted costs showed only modest increases.

China is a large and is still a developing country with 34 provinces/municipalities/regions. While the first tier cities/regions are highly cosmopolitan, like Beijing or Shanghai, there are many less developed areas, more in align with developing countries. Here we showcase Guangxi Zhuang Autonomous Region (Guangxi for short), to exemplify a developing region in China. Located in southwest China, Guangxi is the 9th largest province in China with GDP per capita of $5,865, ranking 17^th^ in its GDP contribution to the Chinese economy in 2015. What also distinguishes Guangxi from other provinces is that it hosts multiple ethnic groups, in particular, Zhuang people account for 33% of its population. This province has beautiful scenery and was less known to the world until recent promotion as a tourist destination and business development zone by the Central government (http://www.gxfao.gov.cn/waishixinxi/xinwenyaowen/2016-05-27/1672.html). Guangxi has over 1,000 hospitals, almost all of which are ranked as primary, secondary and tertiary levels. With each level, hospitals may be ranked as class A and B. While the healthcare system of Guangxi provides treatments for all common diseases, we will limit our evaluation to cardiovascular diseases (CVD) for consistency. Like many countries around world [3, 4], CVD is a major illness in Guangxi, and treatment of this disease is responsible for a significant fraction of healthcare expenditures[5]. Specifically, the prevalence of CVD is high, with 97‰ for hypertension, 22‰ for diabetes, 8 ‰ for heart disease and 5‰ for cerebral vascular diseases, accounting for 42%, 9%, 3% and3%, respectively of all chronic diseases in Guangxi in year 2013 [6].

In this study, we describe the temporal patterns of healthcare costs from hospitalizing patients who suffer from CVD. Such patterns provide empirical insights into current healthcare status of Guangxi from years 2013 to 2016 and directly address whether healthcare costs are on the rise in the Guangxi healthcare system [2, 7]. Further, we intend to identify factors that may associate with costs of hospitalizing CVD patients, specifically, patient’s age, occupation, gender, ethnicity, and payment methods. Understanding the relationship of these factors to healthcare costs may shed light on equity of healthcare delivery and identify potential areas for improving delivery efficiency. Finally, we examine the composition of hospitalization costs and their temporal trends over this period of time. Empirical results and associated conclusions may be helpful for the Guangxi government to assess current healthcare status and to develop future policies to improve the local healthcare delivery system. Lessons learned from Guangxi may also be useful for other comparable provinces in China or other developing countries in the world.

## Materials and methods

### Ethics approval

This study includes hospitalization summery reports (HSR) from over 760,000 hospitalizations. Guangxi Health and Family Planning Commission (GXHFPC) provides de-identified HSR data without patient identifiers, such as security number, name, address, etc.. Further, hospital names are de-identified. This study is approved by Guangxi Medical University Medical Ethic Committee. The Committee also prohibits authors to share the underlying data wholly owned by GXHFPC.

### The data source

This retrospective study utilizes 760,000 HSRs provided by GXHFPC, from January 1, 2013 to May 30, 2016 (“the reporting period” for this study). In Guangxi, GXHFPC manages 67 tertiary hospitals, 232 secondary hospitals, and over 1,000 primary hospitals. Following the guideline of National Health and Family Planning Commission (NHFPC), GXHFPC requires all hospitals to submit HSR to monitor healthcare quality of all public hospitals except maternal and child health hospitals, initially starting data collection from tertiary hospitals in 2013 and from secondary hospitals in 2015. While primary hospitals are not required, some of them also voluntarily submit HSR to GXHPC. Using the standardized HSR form designed by NHFPC (http://www.nhfpc.gov.cn/), each hospital collects HSR on every hospitalization, recording basic demographics, payment methods, admission and discharge dates, admission related information, and discharge related information.

Pertinent to this study are the initial and main diagnoses recorded on HSR, in addition to up to 15 secondary diagnoses. GXHFPC requires using 10^th^ Edition/Revision International Classification of Diseases (ICD-10) codes for all diagnoses. For the current study, we include patients whose initial diagnosis at admission or main diagnosis at discharge are CVD, with first three ICD-10 codes as (I00-I99) [4]. Further, we restrict to those hospitals who have hospitalized at least 100 patients over the reporting period, resulting in 141 hospitals considered for this study. These 141 hospitals include nearly all hospitals in Guangxi that have medical capacities to hospitalize CVD patients.

GXHFPC provided a CVD data set to facilitate this study, but has not provided any funding support for this study, and has no influence on design or conduct of this study or on interpretation of results.

### Outcome variables

#### Total hospitalization costs

Extracted from HSR is the total cost from each hospitalization. All costs are originally recorded in Chinese yuan. To provide more familiar context to international readers, we divide the total cost by 6, which approximates the equivalent US dollar amount and the dollar values should be treated as nominal costs. This total cost is referred to as Cost per Stay (CPS). Together with length of stay (LOS, see definition below), we compute Cost per Day (CPD) by dividing CPS over LOS. It is important to note that both CPS and CPD are meaningful to measure hospitalization expenses. However, when measuring surgical treatments, it is appropriate to use CPS only, whereas CPD becomes less meaningful. Both CPS and CPD have skewed distributions to the right, and thus are transformed by logarithmic transformation with a shift of one, i.e., log(CPS+1) and log(CPD+1), respectively.

#### Composition of hospitalization costs

Also reported on HSR are detailed breakdowns of hospitalization cost. Specifically, total cost includes medical service fees (such as checkups, bed, consultation, etc.), nursing fees (for nursing services at various intensity levels), examination costs (like CT, laboratory tests, etc.), drug costs, and procedure costs (such as injection, debridement, dressing, etc.). The cost breakdown allows us to examine structure of hospitalization expenses and their trends.

#### Length of Stay (LOS)

We define length of stay (LOS) as the difference between admission and discharge dates (including death or transferring to other residential institution). Numerically, it was calculated by discharge date minus admission date. If admission date and discharge date were the same, LOS was set to 1 day.

#### Medical payment methods

All hospitals in Guangxi at this time are public hospitals, and they receive basic financial and infrastructural support from local governments. To cover daily operational expenses, all hospitals collect payments for medical services. There are at least 11 recorded payment methods in HSR. For farmers, nearly all of them are covered by Rural Cooperative Medical Insurance System (NRCMIS) [8]. Urban residents without a stable job are covered by Urban Resident Basic Medical Insurance System (URBMIS) [8]. Employed workers are covered by the Urban Employee Basic Medical Insurance System (UEBMIS) [8]. The Public Health Insurance Program provides payments for retired officials (who started work prior to 1949). For those in exceptional poverty, the government has a Poverty Relief program to cover healthcare costs. Many people pay for their own healthcare expenses, which is referred to as self-insured. In recent years, private commercial insurance companies have emerged to provide basic coverage or to subsidize other insurance programs. HSR records payment method for each hospitalization, and this factor may influence CPS and CPD.

#### Occupations

In China, the majority of Chinese people are accustomed to working in one job for their entire life. Occupation becomes a defining characteristic for an individual in society. For example, a governmental employee tends to be well regarded for their stability. Hence, occupation is an important factor that may associate with hospitalization costs.

### Missing data

The analytic data provided by GXHPC are directly downloaded from HSR database. The database receives hospital-based data directly from individual hospital system, and all data elements are extracted from electronic medical records. Among analytic data variables, there are few missing values, except that the gender variable has one missing value, initial diagnosis variable has 452 missing values, and ethnicity variable has 1,586 missing values. Assuming that these missing values are missing completely at random, our analysis excludes these missing values when corresponding variables are included in the analysis.

### Statistical analysis

We summarize baseline characteristics of the study hospitals and associated hospitalized patients by tabulating frequencies and percentages for categorical variables and discretized continuous variables. We use generalized additive models (GAM) [9] to describe the temporal changes of CPS and CPD from years 2013 to 2016. In the data analysis, we regress the outcome on discharge dates by GAM, i.e., without imposing a specific outcome function of calendar time. The GAM regression allows us to capture any temporal trends of outcome variable over time. In notation, the GAM model may be written as

$$CPS_{i}(t_{i})=s(t_{i})+\varepsilon_{i}\;,$$

where *CPS*_*i*_(*t*_*i*_) represents the CPS for the ith visit at time *t*_*i*_, *s*(*t*_*i*_) is a non-parametric function of time t, and *ε*_*i*_ is a random variation. Given our primary goal to estimate the temporal trend, we have adjusted other covariates. Similar models are fitted for CPD and LOS.

For analytic assessment of potential associated factors, we use generalized linear model (GLM) to measure outcome association with the potential factor, after adjusting for hospital-to-hospital heterogeneities between 141 hospitals and four calendar years [10]. Specifically, we introduce 141 indicator variables for corresponding hospitals and 4 indicator variables for corresponding calendar years, and include them in relevant regression analyses, to adjust for hospital-specific and calendar-specific heterogeneities. In notation, the GLM model may be written as

$$CPS_{i}=\beta \prime x_{i}+\sum_{j\;=\;1}^{141}\alpha_{j}I_{j}(H_{i})+\;\sum_{k\;=\;2014}^{2016}\gamma_{k}I_{k}(C_{i})+\varepsilon_{i}\;,$$

where $\sum_{j\;=\;1}^{141}\alpha_{j}I_{j}(H_{i})$ is a linear combination of indicators modeling hospital-to-hospital heterogeneity, $\sum_{k\;=\;2014}^{2016}\gamma_{k}I_{k}(C_{i})$ is a linear combination of indicators modeling calendar-to-calendar heterogeneity, and *β*′*x*_*i*_ includes the regression component with covariates of interest.

We used R software version 3.3.0 (http://www.r-project.org/), a commonly used statistical package in the public domain, to carry out all statistical analyses, including tabulations, drawing figures, and regression analysis.

## Results

### Study hospitals

After filtering hospitals with fewer than 100 hospitalizations during the reporting period, we netted a total of 141 hospitals for this study, including 39 tertiary hospitals, 91 secondary hospitals, 6 primary hospitals and 7 hospitals with unknown ranking (see S1 Table). Note that one hospital, with unknown ranking, was assigned a secondary hospital ranking during the reporting period. Similarly, two secondary hospitals under evaluation were assigned a tertiary hospital status (hence these three hospitals are counted twice in S1 Table).

### Study population

Table 1 shows the characteristics of all hospitalized patients in 141 hospitals during the reporting period. Among 760,000 CVD hospitalizations, 329,496 are from tertiary hospitals, 416,215 from secondary hospitals, 2,782 from primary hospitals and 11,507 from unranked hospitals. Here the sampling unit is the hospitalization, rather than patient. It is possible that some patients have multiple hospitalizations that can not be differentiated. Among all hospitalizations, female patients accounted for 43.9% and males accounted for 56.1%.

**Table 1:** Distributions of all hospitalized patients across different levels and classes of hospitals, with respect to gender, age, marital status, ethnicity, and year of hospitalizations under the study.

Level	Primary	Secondary			Tertiary			UNS*	Total
Class		A	B	UNK*	A	B	UNK*		
**Sample Size**=	2,782	398,924	13,322	3,969	275,976	23,643	29,877	11,507	760,000
**Sex**									**Percentage**
Female	48.5	42.8	43.4	46.1	40.5	42.4	43.6	43.0	43.9
Male	51.5	57.2	56.6	53.9	59.5	57.6	56.4	57.0	56.1
**Age**									
<18	8.4	2.5	2.1	0.6	1.5	0.9	1.2	4.7	2.6
18-	4.6	4.5	4.8	5	5.6	4.4	6.1	7.2	4.8
40-	6.4	8.2	8.1	8.1	9.6	8.6	9.4	8.6	8.0
50-	11.8	15.9	13.8	15.4	18.6	17.3	18.4	14.2	15.4
60-	21.1	25.4	25.1	25	26.3	27.3	26.5	23.5	25.4
70-	28.1	28.1	30.2	28.4	24.2	25.8	25.1	26.3	27.4
80-	19.6	15.4	15.9	17.5	14.2	15.6	13.3	15.5	16.3
**Marital Status**									
Married	73.6	82.3	83.5	90.7	87.5	91.3	90.7	85.9	87.1
Single	9.6	8.5	4.6	3.9	3.6	3.2	3.0	8.8	5.1
Other/Unkown	16.9	9.2	11.8	5.4	9.0	5.5	6.3	5.3	7.7
**Ethnicities**									
Han	83.4	66.3	31.4	69.6	87.9	98.3	78.6	69.9	76.2
Zhuang	15.1	29.2	63.0	27.4	10.4	1.1	20.7	27.5	21.6
Other Ethnicities	1.5	4.5	5.6	3.0	1.7	0.5	0.8	2.6	2.2
**Year of Hospitalization**									
2013-	12.8	14.4	15.1	7.6	14.4	14.6	13.6	8.9	14.3
2014-	32.6	40.0	36.7	43.4	37.7	51.0	36.4	40.8	41.8
2015-	54.2	41.5	42.9	48.6	41.8	34.2	40.6	49.9	41.0
2016- (up to May, 31)	0.4	4.1	5.3	0.3	6.0	0.2	9.5	0.4	2.9

* UNS- unspecified, UNK- unknown

With respect to patients’ age distribution, older patients (60 years or older) account for over half of the hospitalizations (58.7%). There are 2.6% of all hospitalizations for younger patients than 18 years old.

Most hospitalized patients are married (87.1%). With respect to distribution of ethnic groups, Han Chinese account for 76.2%, followed by the largest minority Zhuang Chinese with 21.6%. There are other ethnic groups, which combined account for 2.2%. Examining this distribution across different hospital types, it is noticeable that appreciable percentages of Zhuang patients are hospitalized in secondary hospitals, and disproportionally large percentage of Han patients are hospitalized in tertiary hospitals. With respect to years of HSR collection here, it is clear that most are collected in 2014 and 2015 (41.8% and 41.0%, respectively), while year 2013 is the starting year, and year 2016 has included only five months.

### Temporal trend of costs

**Fig 1:**
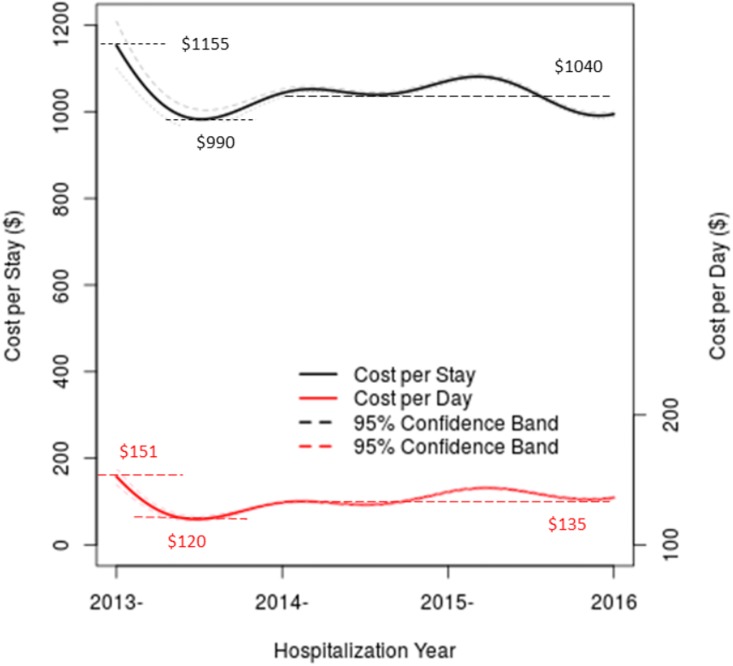
Temporal trends of cost per stay indicated by the left y-axis (from $0 to $1,200), and temporal trends of cost per day indicated by the right y-axis (from $50 to $250).

Fig 1 shows CPS from year 2013 to 2016, with its value indicated on the left y-axis. In contrast to rapid rise of CPI in China [2], local CPI in Guangxi is relatively stable according to official statistics provided by Guangxi Statistical Bureau (http://www.gxtj.gov.cn/). For this reason, we choose not to adjust CPI in computing CPS. Similarly, CPD is computed without correcting for CPI. Fig 1 shows a decline of CPS from $1,155 to about $990 in the first half year of 2013. Then, the CPS bounces back to an average of $1,040 afterwards. It appears that CPS has a small declining trend in the middle of year 2015. When stratifying over tertiary and secondary hospitals, we have shown that the costs in the secondary hospitals (red line) and the tertiary hospitals (blue line) also have a sharp decline in the early year of 2013 (S1 Fig). In tertiary hospitals, the average CPS starts at $1,750 in year 2013, decreases sharply to $1,300 in the mid part of 2013, and afterwards, it has two escalations from $1,300 to $1,650 between mid-2013 and early 2014 and from $1,500 to $1,750 between mid-2014 and early 2015, and it decreases to $1,300 in 2016. Similarly, CPS in the secondary hospitals starts at ~$700 in year 2013, sharply decreases to $550 in mid-2013 and then bounces back to around $800. Since the overall temporal pattern remains comparable, we treat all hospitals equally in the overall assessment of healthcare costs.

Fig 1 also shows the temporal trend of CPD, indicated with y-axis on the right hand side. Mimicking the temporal pattern of CPS, CPD appears to have the first sharp decline due to the same artifact. Beyond year 2014, CPD appears to be stabilizing around $135 throughout the rest of the reporting period.

### Composition of hospitalization costs

**Fig 2:**
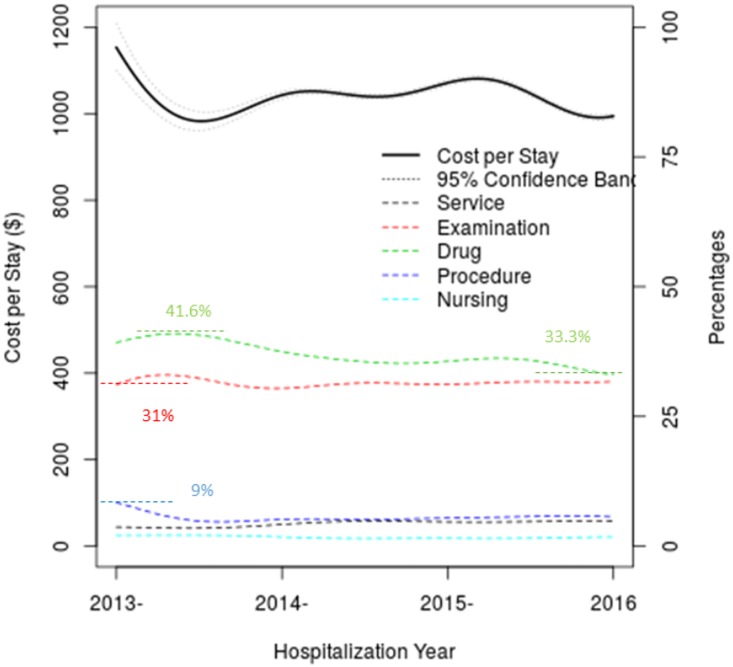
Compositions of cost per stay indicated by the left y-axis (from $0 to $1,200), and percentages of specific costs (services, examination, drug, procedure and nursing) by the right y-axis (from 0% to 50%).

In the context of total hospitalization cost for each hospital stay, Fig 2 shows detailed breakdown of CPS by five different major categories: service, procedures, nursing, examination and drugs. While confidence bands are shown for the overall CPS, they are included because clouding cost curves. Percentages of costs are indicated by y-axis on the right hand side. Visually, the drug cost, a major expense, takes more than 1/3 of CPS. Interestingly, the drug cost appears to decline gradually, from 41.6% to 33.3% of CPS, during the reporting period. The second major component of CPS is associated with examination cost. It appears that the examination cost is stable around 31%. Both drug and examination costs appear to converge to 33% of CPS. In contrast, service, procedure and nursing costs appear to be quite modest, and are generally less than 9%, about 1–2%, 5% and 6%, respectively. Other costs, not listed in the figure, include surgical costs, material costs, traditional Chinese medicine and rehabilitation.

### CPS of surgeries

**Fig 3:**
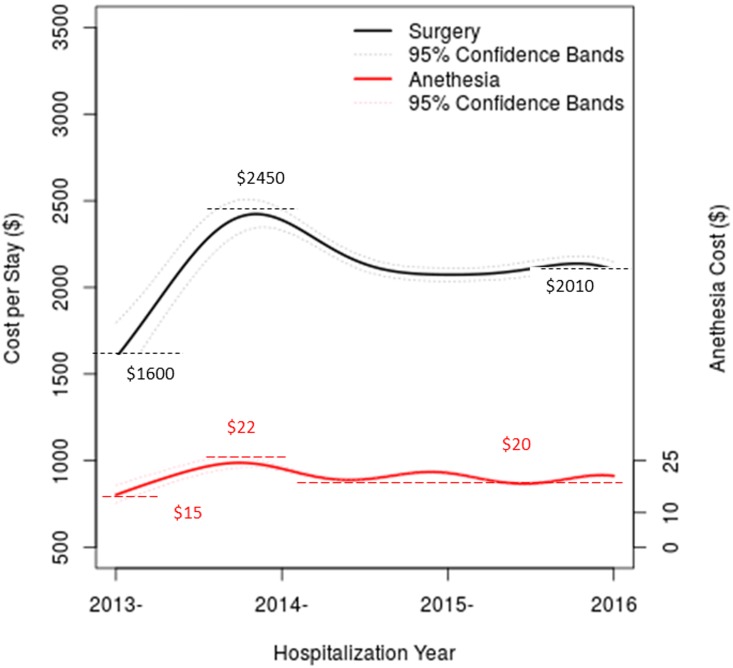
Temporal trends of surgery-specific cost per stay indicated by the left y-axis (from $0 to $2,500), and temporal trends of anesthesia cost indicated by the right y-axis (from $0 to $40).

Among 760,000 hospitalizations, 183,546 hospitalizations include surgeries or surgical interventions, and are thus used to evaluate surgery-specific CPS. Fig 3 shows the temporal trend of surgery-specific CPS, together with its confidence bands, the value for which is indicated by y-axis on the left hand side. It appears that the surgical CPS sharply increases in year 2013–2014 from $1,600 to $2,450. Then, surgery-specific CPS has a small adjustment, and stabilizes around $2,100, for the rest of the reporting period. In contrast, anesthesia cost increases modestly from $15 to$22, when surgery cost shows a major increase. After year 2013, the anesthesia cost becomes stable with a small fluctuation around $20.

### Length of Stay (LOS)

**Fig 4:**
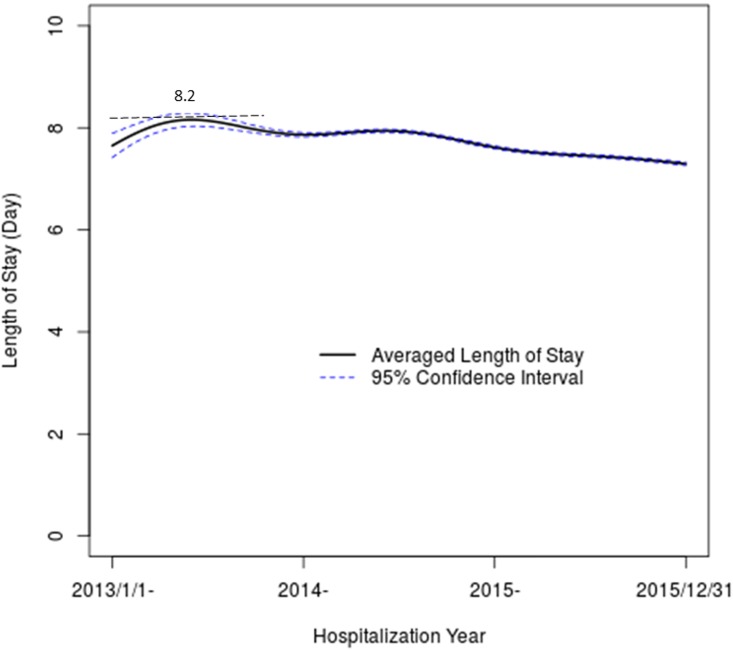
Temporal trends of Length of Stay (LOS) estimated for all hospitalizations of CVD patients, with their 95% confidence bands.

While primary outcome variables are hospitalization costs, LOS remains an important variable, since it is used in computing CPD, and it is an index that measures efficiency of healthcare delivery. Fig 4 shows temporal trend of LOS over the reporting period, with 95% confidence bands. It appears that LOS has a gradual declining trend from 8.2 days down to 7.5 days, from year 2013 to year 2016. This gradual decline in part explains the declining CPS, while CPD remains constant (Fig 1).

### Factors associated with CPS and CPD

Recognizing that CPS and CPD reflect two different aspects of hospitalization costs, we treat them as two separate outcomes, and explore factors that may associate. All analyses use GLM to estimate regression coefficients after adjusting for heterogeneity between 141 hospitals and 4 calendar years. Table 2 presents coefficient, standard error, Z-score and p-value in left and right panels. Treating female patients as the reference group, the estimated coefficients 0.00468 and 0.00367 for the male patients, associated with CPS and CPD, respectively, are significantly greater than zero (p-value<0.001). These results indicate that CPS and CPD of hospitalizing male patients are significantly higher than those of hospitalizing female patients. To place the estimated coefficient into a familiar scale, we convert the coefficient into an absolute scale in dollars. Suppose that treating a female patient costs $999. Equivalently, the cost of treating a male patient approximates $10^{0.00468+{\text{log}}_{10}(999+1)}-1\approx 1009.8$. In other words, hospitalizing a male patient costs about $1,011, i.e., $11 more than hospitalizing a female patient on average. While the average difference is modest, the p-value is highly significant because of large sample sizes.

**Table 2:** Assessing associations of cost per stay and cost per day with sex, age, marital status, and ethnicity through estimating coefficient, standard error, Z- score and P-value, with an adjustment across all 141 hospitals and 4 calendar years.

	Cost per Stay^1^				Cost per Day			
	Coef^2^	SE	Z-score	P-value^3^	Coef	SE	Z-score	P-value
**Sex (reference is female)**								
Male	4.68E-02	9.72E-04	4.81E+01	<1.0E-99	3.67E-02	8.23E-04	4.46E+01	<1.0E-99
**Age (reference is 70–79)**								
<18	-3.54E-01	3.78E-03	-9.35E+01	<1.0E-99	-2.01E-01	3.22E-03	-6.25E+01	<1.0E-99
18-	-9.41E-02	2.36E-03	-3.98E+01	<1.0E-99	1.98E-02	2.01E-03	9.84E+00	7.46E-23
40-	-2.16E-02	1.88E-03	-1.15E+01	1.18E-30	4.27E-02	1.60E-03	2.67E+01	<1.0E-99
50-	-4.38E-03	1.50E-03	-2.92E+00	3.46E-03	3.38E-02	1.27E-03	2.65E+01	<1.0E-99
60-	5.44E-03	1.33E-03	4.09E+00	4.36E-05	2.06E-02	1.13E-03	1.82E+01	3.40E-74
80-	-6.52E-04	1.56E-03	-4.18E-01	6.76E-01	-5.68E-03	1.33E-03	-4.27E+00	1.93E-05
**Marital Status (reference is married)**								
Single	-1.35E-01	2.38E-03	-5.67E+01	<1.0E-99	-7.14E-02	2.02E-03	-3.53E+01	<1.0E-99
Other situations	1.24E-04	2.13E-03	5.83E-02	9.54E-01	-3.83E-03	1.81E-03	-2.12E+00	3.43E-02
**Ethnicity (reference is Han)**								
Zhuang	5.23E-03	2.15E-03	2.43E+00	1.52E-02	-1.23E-03	1.79E-03	-6.87E-01	4.92E-01
Other Ethnicities	6.60E-03	3.77E-03	1.75E+00	7.98E-02	1.47E-03	3.13E-03	4.69E-01	6.39E-01

^1)^ Costs are transformed to the logarithmic scale with a location shift of one

^2)^ adjusted for heterogeneity associated with 141 hospitals and four calendar years

^3)^ Highly significant p-values are highlighted by yellow mark

The second factor under consideration is age of patient when being hospitalized. Because of relatively high frequency, we use patients aged 70–79-year-old as the reference group. With respect to CPS, the cost for hospitalizing a patient aged 80 years or older is comparable to the reference group. It is interesting that the cost for a bit younger patient (60–69 years old) is significantly higher (p-value<0.001). For other four younger age groups, CPS is proportionally less. In contrast, CPD is relatively higher for patients aged 18 to 69, while CPD is less for very young and very old patients. While consistent CPD and CPS for a particular age group are within expectations, it is interesting to note that CPD and CPS for age groups 18–39, 40–49 and 50–59 are opposite: higher CPD but less CPS. These results may indicate that these patients are at their prime ages with better recoveries, or physicians may offer more active treatment plans for these patients so that they can leave hospitals as soon as possible back to work.

With respect to marital status, we treat married patients as the reference group. It appears that both CPS and CPD are significantly less for single patients (p-value<0.001), probably because the majority of single patients are younger people. For other marital status, including divorced patients, CPS and CPD are largely comparable.

As noted earlier, the presence of multiple ethnic groups, in particular, Zhuang Chinese, is a unique characteristic for Guangxi. For CPS and CPD, it appears that there are not much differentiations between minority and Han Chinese, with an exception for CPS among Zhuang Chinese (p-value = 0.0152). Even for this significant difference, the absolute difference in CPS is relatively small, and equals $10^{0.000523+{\text{log}}_{10}(999+1)}-1\approx 1000.21$, i.e., on a $999 CPS paid by a Han Chinese, Zhuang Chinese would pay on average $1.21 more.

### Payment methods and costs

In China, the most common form of insurance is UEBMIS for urban workers, provided through their employers, and may be considered as a well-accepted standard. Treating this program as a reference, Table 3 lists differences of CPS and on CPD for other insurance programs, along with their frequencies (the first column). Among these insurance programs, NRCMIS was recently developed to cover all farmers in the countryside who have never had any form of insurance in the past, and now covers over 249,000 hospitalizations during the reporting period. It is interesting to note that CPD associated with this program is significantly higher than the reference, while CPS is substantially less. For the insurance program for urban residents (URBMIS), CPS and CPD present a similar discordant pattern, as does the self-insurance program.

**Table 3:** Estimated cost per stay and cost per day across different payment methods, where insurance for urban worker is treated as the reference.

Payment Method	Frequency	Cost per Stay^1^				Cost per Day			
		Coef^2^	SE	Z-score	P-value^3^	Coef	SE	Z-score	P-value
UEBMIS	152,113	3.17E+00	4.54E-03			2.27E+00	3.88E-03		
NRCMIS	249,124	-1.09E-02	1.60E-03	-6.84E+00	7.75E-12	3.69E-02	1.36E-03	2.71E+01	2.27E-161
Self-Insruance	69,033	-3.75E-02	2.10E-03	-1.79E+01	2.35E-71	5.84E-02	1.79E-03	3.26E+01	1.31E-232
URBMIS	34,420	-1.86E-02	2.66E-03	-6.99E+00	2.66E-12	1.39E-02	2.27E-03	6.13E+00	8.68E-10
Public Health Insurance	9,873	2.10E-02	4.80E-03	4.38E+00	1.17E-05	2.70E-02	4.10E-03	6.60E+00	4.13E-11
Other Insurance	3,888	5.86E-03	7.06E-03	8.29E-01	4.07E-01	2.59E-02	6.04E-03	4.29E+00	1.83E-05
Poverty Relief	344	3.37E-02	2.32E-02	1.45E+00	1.46E-01	2.40E-02	1.98E-02	1.21E+00	2.26E-01
Commercial insurance	195	-6.91E-02	3.24E-02	-2.13E+00	3.31E-02	-3.44E-02	2.77E-02	-1.24E+00	2.15E-01
Other Types	113,608	-1.96E-03	3.55E-03	-5.53E-01	5.81E-01	2.47E-02	3.04E-03	8.14E+00	3.88E-16

^1)^ Costs are transformed to the logarithmic scale with a location shift of one

^2)^ adjusted for heterogeneity associated with 141 hospitals and four calendar years

^3)^ Highly significant p-values are highlighted by yellow mark

### Occupations and cost

Treating government employee as the reference group we assess whether CPS and CPD for other occupations are comparably lower or higher. Table 4 lists 12 occupations to compare their CPS and CPD with government workers. The single largest occupation is the farmer with over 337,000 hospitalizations, who appear to have higher CPS and CPD (p-value<0.001). The second largest group is retirees, and their CPD seems to be significantly less than the reference (p-value<0.001), while their CPS are significantly higher than the reference group (p-value<0.001).

**Table 4:** Estimated cost per stay and cost per day across different occupations, where government employee is treated as the reference.

Occupations	Frequency	Cost per Stay^1^				Cost per Day			
		Coef^2^	SE	Z-score	P-value^3^	Coef	SE	Z-score	P-value
Government Employee (Reference)	11,017	3.15E+00	5.86E-03			2.29E+00	4.97E-03		
Farmer	337,114	1.55E-02	4.38E-03	3.53E+00	4.11E-04	2.45E-02	3.71E-03	6.61E+00	3.85E-11
Retiree	149,064	3.04E-02	4.45E-03	6.84E+00	8.07E-12	-2.38E-02	3.77E-03	-6.32E+00	2.65E-10
Unemployed	25,411	-3.39E-02	5.15E-03	-6.59E+00	4.26E-11	-7.02E-03	4.36E-03	-1.61E+00	1.07E-01
Worker	20,277	2.02E-02	5.37E-03	3.77E+00	1.61E-04	6.15E-03	4.55E-03	1.35E+00	1.76E-01
Freelancer	19,475	-2.49E-02	5.69E-03	-4.38E+00	1.18E-05	1.63E-02	4.82E-03	3.37E+00	7.39E-04
Office Worker	15,976	-1.77E-02	5.30E-03	-3.35E+00	8.13E-04	2.05E-03	4.49E-03	4.57E-01	6.48E-01
Technical Professional	6,172	7.93E-03	6.86E-03	1.16E+00	2.48E-01	2.33E-02	5.81E-03	4.01E+00	6.04E-05
Self-Employed	5,531	-6.36E-03	7.08E-03	-8.99E-01	3.68E-01	3.10E-02	6.00E-03	5.17E+00	2.30E-07
Student	5,124	-2.65E-01	7.25E-03	-3.65E+01	3.39E-292	-1.22E-01	6.14E-03	-1.99E+01	7.68E-88
Manager	876	-3.78E-02	1.48E-02	-2.56E+00	1.05E-02	-2.00E-02	1.25E-02	-1.60E+00	1.10E-01
Solider	280	-1.21E-01	2.54E-02	-4.76E+00	1.96E-06	6.28E-03	2.15E-02	2.92E-01	7.71E-01
Others	162,539	-1.22E-02	4.43E-03	-2.76E+00	5.79E-03	4.63E-04	3.75E-03	1.23E-01	9.02E-01

^1)^ Costs are transformed to the logarithmic scale with a location shift of one

^2)^ adjusted for heterogeneity associated with 141 hospitals and four calendar years

^3)^ Highly significant p-values are highlighted by yellow mark

## Discussion

We utilize the “Big Administrative Data” of HSRs from 141 hospitals to identify 760,000 patients hospitalized for CVD from early 2013 to May 2016, in Guangxi Zhuang Autonomous Region, People’s Republic of China. To the best of our knowledge, this study is among the largest big data investigation from China, and the study population is from a developing region, as opposed to developed regions like Beijing or Shanghai in China. Therefore, observations and tentative conclusions derived from this investigation shed new insights into the Chinese healthcare system. Globally, some results may be more closely in alignment with those from other developing countries.

### Rising costs of healthcare?

Popular opinion in China is that medicine becomes ever more expensive (e.g., the popular Chinese phrase “看病贵”). Even though this perception is initiated in the first tier cities, like Beijing and Shanghai, it permeates the entire country through mass media and social-media on internet. Indeed, such a perception inevitably creates enormous pressure on healthcare systems throughout China, and it can sometimes be a source of social instability. When examining CPS and CPD of hospitalizing CVD patients in Guangxi, we found that costs of hospitalizing CVD patients are relatively stable over the reporting period (Fig 1). In fact, the noticeable trend, observed from CPS and CPD, is that the hospitalization costs for tertiary hospitals are stable in the first half of year 2013, followed by fluctuating but flat trends. If anything, CPS appears to have a downward trend from the middle of year 2015, while CPD remains stable. The observed trends may have resulted from implementing healthcare policies. For example, the Chinese government implemented a new policy to reduce drug prices for over 400 different medications by as much as 15% in the early part of year 2013. Among the 400 medications, 16 are CVD related drugs and the price of Cilnidipine Capsules, the most commonly used medication for reducing blood pressure, decreased from$3.42 to $2.04 with a reduction of 40%.The price of Voglibose Capsules, the most commonly used medication for reducing blood sugar, decreased from $5.62to $4.03 with a reduction of 28%. During the implementation of this national healthcare reform policy, Guangxi selected 6 secondary hospitals (at county level) in year 2011 to participate in the national pilot program that imposes zero markup on drug costs, and then gradually broadened the pilot program to all county-level secondary hospitals by year 2015 (http://www.gxhfpc.gov.cn/). Additionally, Guangxi has encouraged using inexpensive generic medications since year 2012 and intensified this to a requirement in year 2015 (http://www.gxhfpc.gov.cn/). Besides influence from governmental policies, insurance agencies (different governmental agencies, typically controlled by central government) have promoted new medical insurance policies, aiming to control CPS and LOS in combination with adopting standard clinical pathways and rational use of medications [11, 12], in addition to control “annual global budget” [13, 14]. On the other hand, the fluctuations of CPS reflect the reimbursement policy of medical insurance, i.e. the hospitals are reimbursed once a year. Before receiving funding, insurance agencies conduct audits. In response, there is a tendency to closely monitor CPS, in compliance with the requirement of medical insurance polices, resulting in periodic fluctuations, especially among tertiary hospitals.

### Rising surgical cost?

In contrast to the overall CPS, surgery-specific CPS appears to experience a sharp increase in years 2013 to 2014, from $1,600 to $2,450, i.e., a whopping increase of over 50%. In the following year, the surgery-specific CPS reduces to around $2,010. This rising surgery-specific CPS is mostly associated with the introduction of new surgical techniques and procedures, as new surgical technologies are significantly improving quality of health care. However, from the patient’s perspective, this may feed the perception that healthcare costs are rising rapidly.

### Compositions of CPS

Hospitalizing a patient is a team activity requiring support from physicians, nurses, lab technicians, medical technologists, pharmacists and administrative staff. In the current analysis, we consider five components: service, examination, drug, procedure, and nursing, and we present their temporal trends (Fig 2). Drug costs have a major impact on CPS [15]. As shown, drug cost appears to be steadily declining from 42% down to 33%, over years 2013 to 2016. This gradual decline is probably associated with newly implemented healthcare reform policies that aim to increase use of generic drugs to control drug costs.

Within CPS, service cost covers mostly physicians’ service, while the nursing fees account for nursing services. From our analysis, one can see that both physicians’ and nurses’ services are relatively small components in CPS, a unique characteristic of the healthcare system in China. Recognizing that healthcare services are highly complex and involve skilled workers, one wonders how to structure healthcare to provide appropriate compensation incentives for healthcare workers, so that these healthcare providers are motivated to provide best care to patients. Of course, appropriate incentives are also important to encourage young people to acquire medical educations and to become healthcare providers in the future.

### Factors associated with CPS and CPD

Strictly speaking, CPS and CPD are largely dictated by disease diagnosis and related treatment. In practice, other factors may associate with CPS and CPD. Identifying such factors helps us to understand nature of hospitalization expenses, and may also help us to identify opportunities to improve healthcare delivery efficiency. Specifically, we found that Guangxi hospitals treat fewer female patients (44.6%) than male patients (55.4%), which is in line with earlier reports in China [5, 16–18]. Both CPS and CPD are significantly higher for male patients than for female patients (p-value<0.001). One possible explanation is that Chinese men have higher incidence of CVD than Chinese women because of their drinking and smoking life style [19, 20]. Indeed, it is reported that in United Kingdom, prevalence rates of chronic heart failure in men and women are 6.4% and 4.9%, respectively [21]. There is potentially an alternative explanation. In Guangxi, men tend to be the majority in the countryside workforce, so there may be some biases favoring hospitalizing men, while female patients tend to receive care outside of the hospitals. Such a phenomenon is certainly different from observations in United States, where women are more attentive to health care issues, e.g., female patients accounted for 55.7% in heart failure hospitalizations [22].

Our analysis suggests that the patient’s age is another important factor associating with CPS and CPD. When examining distribution of patient’s age, it is noted that there is an obvious deficit of patients older than 80 years, even though the distribution for patients younger than 80 is comparable to those reported in China [23] and in United States [3]. While life expectancy may be one possible explanation for this deficit [24], it may also associate with local Guangxi tradition. Older patients in the countryside often avoid hospitalization because they fear being cremated if they die in the hospital, and they prefer traditional burial practices [25]. Now with respect to CPD, the daily costs are higher for hospitalizing patients from age 40 to 69 years old, in comparison with age group 70–79 years old. Treating youngest and oldest groups cost less than of treating 70–79 years old. Interestingly, from the perspective of CPS, treating patients 40–59 years old tends to be less, probably because younger patients recover faster and are eager to go back to work.

Ethnic diversity is a hallmark of Guangxi, with a major ethnic group of Zhuang Chinese. In our analysis, Zhuang patients account for 20.6% of all hospitalizations. Proportionally, this percentage is smaller than its population percentage of 33% in Guangxi. Noticeably, disproportionally large percentages of Zhuang patients are hospitalized in secondary hospitals, especially in Secondary B hospitals with 63%. This observation suggests that Zhuang patients tend to seek care in primary and second hospitals, which is in align of government policy seeking care in local hospitals. Meanwhile, this observation may also suggest the need to improve the accessibility of tertiary hospitals for Zhuang Chinese. For example, government may strategically build new tertiary hospitals that closer to regions where Zhuang and other minority Chinese live. With respect to CPS and CPD, there is nearly no difference between Han and other minority groups.

### Length of stay

Our analysis suggests that LOS appears to be a gradually declining trend, from the peak of 8.2 days to 7.5 days. This result is largely consistent with the earlier report [18].

### Payment methods

As China is adopting market-driven economy, the country is also trying to diversify from a single payer to multiple payer systems with more insurance programs. Each insurance program, government-owned or private, focuses on its group of recipients, including UEBMIS for urban worker, URBMIS for urban resident (without regular job), and NRCMIS for farmers. All insurance programs use their chosen mechanisms for revenue collection, risk pooling, the benefit package, and provider payment [8]. In comparison with UEBMIS, both URBMIS and NRCMIS have lower CPS but higher CPD (p-value <0.001), probably because both programs push hospitals to control CPS with shorter LOS. To recoup some loss of revenues, hospitals may accelerate treatment schedules, resulting in high CPD with shorter LOS.

Among all payees, hospitalizations paid by commercial insurance appear to have lower CPS and CPD, which may be surprising to many western readers [25]. It is important to note that commercial insurance is new in China, and tend to have relatively low payouts. Certainly, as commercial insurance takes hold in China, more people will likely purchase additional insurance to supplement government-sponsored insurance. As reported earlier, Public Health Insurance pays higher CPS and CPD [26] since patients need little out-of-pocket copayment. By self-insuring, these patients pay the lower CPS but higher CPD, because they want to control the total hospitalization costs by shorter stay. As one can see, payment method is a major factor that affects costs of hospitalizing CVD, which is consistent with an earlier report [27].

### Occupations

Our analysis has shown that occupation is significantly associated with CPS and CPD, which is different from an earlier report [27]. In comparison with government employees, office workers have significantly lower CPS (p-value<0.001), while CPD is comparable between two occupations (p-value = 0.648). This observation indicates that governmental employees probably stay in hospitals a bit longer, possibly because of better job-related benefits. Farmers, on the other hand, appear to have higher CPS and higher CPD in comparison with government employees (p-value <0.001). One possible explanation is that farmers tend to have more severe CVD diseases that result in high CPD and CPS.

### Limitations

While we are enthusiastic about conducting research using big administrative databases of HSRs, we recognize the limitations associated with such big data analytics. HSRs are collected for administrative purposes, and hence are not comprehensive in collecting all “important etiological variables”. Inevitably, our analysis is limited to available data. Secondly, this administrative database captures all hospitalizations in Guangxi, with many cost variables, patient demographics, and clinical outcome variables. Such observational data are useful for assessing associations between variables, but are not appropriate for making any causal inferences. Thirdly, because HSRs are administratively collected data, we have limited control over data quality. For example, some variables may have larger percentages of missing data, and others may have coding errors. Hence, our analysis uses statistical approaches in the hope that large sample sizes would overcome these data deficiencies when producing meaningful results.

## Conclusion

Through analyzing “big administrative data”, we have demonstrated that healthcare cost of hospitalizing CVD in Guangxi is relatively stable and not subject to the popularly perceived “rising healthcare cost”. To examine temporal fluctuations in details, CPS has been stable from year 2013 to year 2014 and appears to have a downward trend in the beginning of year 2016. On the other hand, surgery-specific CPS has experienced some noticeable rise in year 2013, but has since adjusted to equilibrium. Within CPS, drug costs are more than one third of CPS, and, fortunately, are gradually declining over years 2013 through 2016. The analysis of cost composition clearly shows that compensation for physicians and nurses, two major healthcare work forces, are only small fractions of total costs. The LOS of hospitalizing CVD patients is gradually declining from 8.2 days to 7.5 days, indicating the improving delivery efficiency. After adjusting heterogeneity across hospitals and calendar years, we have shown that age, sex, marital status, occupation, and payment methods are significantly associated with CPS or CPD of hospitalization cost of CVD patients. Interestingly, ethnicity does not appear to associate with CPS or CPD, but ethnic minority access to tertiary hospitals seems to be less than Han Chinese’s access.

It is recognized that big data analytics can produce data-driven evidence about current health economics, and evidence may be useful for major stakeholders to evaluate existing policies and to guide the development of new policies. First, leadership in charge of Guangxi healthcare system seems to manage healthcare costs quite well, despite perceived rising healthcare costs around the country. Second, Guangxi government may want to examine allocations of tertiary hospital resources to improve healthcare services to under-served populations. Third, it is advisable for older patients to receive more timely care, so that Guangxi people can live even longer. The Chinese Central government wants to open Guangxi to the world and promote tourism to the region, and to make it the front door to Southeast Asian countries; it is hoped that Guangxi government can maintain its stability and provide affordable healthcare to its people, while improving health care quality. Utilizing big data analytics, the Guangxi government can actively monitor the status of its healthcare system in real time, and it can continuously improve the performance of its healthcare system by focusing on patient-centered outcomes.

## Supporting information

S1 Fig Cost trend over year 2013 to 2016 by hospital levels. (TIF)

S1 Table Distribution of all 141 hospitals in guangxi province with respect to level and class. (XLSX)
