# Supplementary figures and images for: Assessment of potential factors associating with costs of hospitalizing cardiovascular diseases in 141 hospitals in Guangxi, China
Source: PLoS One. 2017 Mar 16;12(3):e0173451. doi: 10.1371/journal.pone.0173451 (PMC5354288; doi:10.1371/journal.pone.0173451)

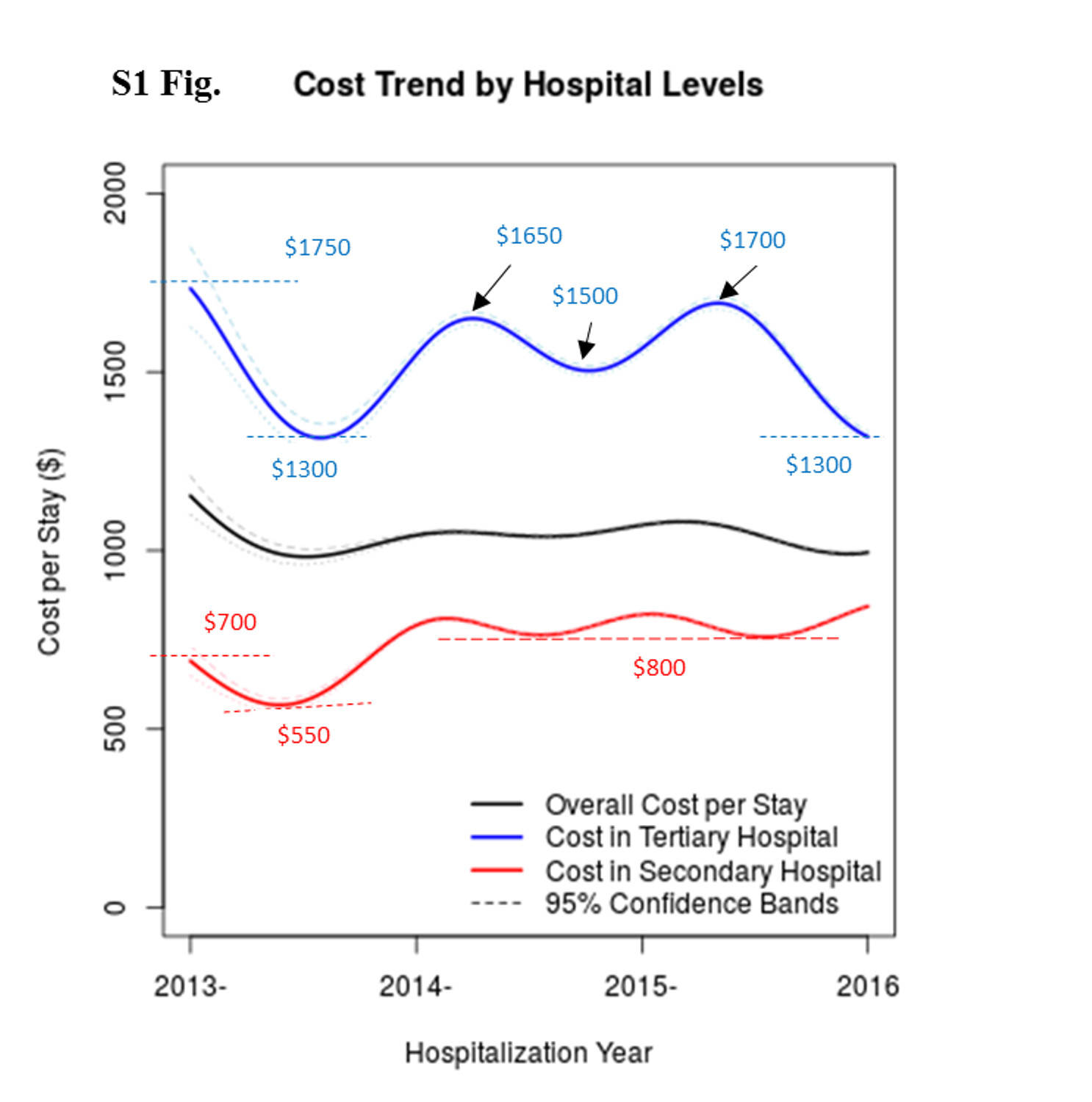

Supplement: S1 Fig — (TIF) [file pone.0173451.s001.tif]
